# Supplementary material for: Generation of a Model to Predict Differentiation and Migration of Lymphocyte Subsets under Homeostatic and CNS Autoinflammatory Conditions
Source: Int J Mol Sci. 2020 Mar 17;21(6):2046. doi: 10.3390/ijms21062046 (PMC7139518; doi:10.3390/ijms21062046)
Supplement: Supplementary file 1 [file ijms-21-02046-s001.pdf]

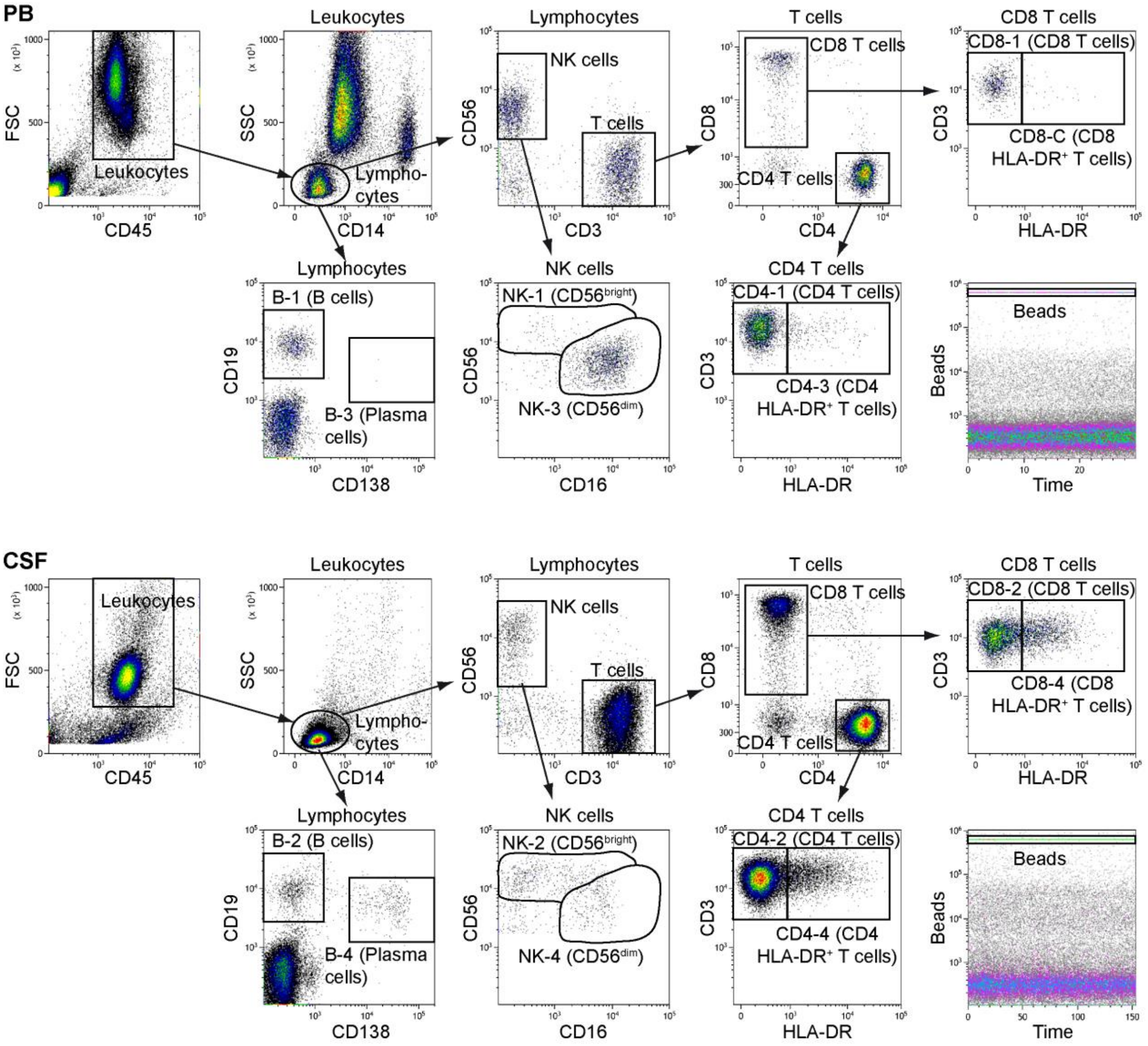

Supplemental Figure 1: Gating strategy. Flow cytometry data of an RRMS patient are shown as an example to demonstrate the gating strategy used to determine the relative proportions of naïve and differentiated lymphocyte subsets in the PB (**top**) and CSF (**bottom**). In a first step CD45$^+$ leukocytes were selected in a CD45 vs. forward scatter channel (FSC) plot. CD45$^+$ cells were then displayed in an CD14 vs. sideward scatter channel (SSC) plot and CD14$^-$/SSC^{low} lymphocytes were selected and displayed in a CD3 vs. CD56 plot to distinguish between CD3$^-$CD56$^+$ NK cells and CD3$^+$CD56$^-$ T cells. T cells were further split into CD4$^+$ T-helper cells and CD8$^+$ cytotoxic T cells in a CD4 vs. CD8 plot. CD4$^+$ T cells and CD8$^+$ T cells were further displayed in an HLA-DR vs. CD3 plot to distinguish between HLA-DR$^-$ naïve and HLA-DR$^+$ differentiated CD4$^+$ and CD8$^+$ T-cell subsets, respectively. Naïve CD19$^+$CD138$^-$ B cells were distinguished from differentiated CD19^{low}CD138$^+$ plasmacytoid cells in a CD138 vs. CD19 plot. Finally, naïve CD56^{bright} and differentiated CD56^{dim} NK cells were distinguished in a CD16 vs. CD56 plot.

		Controls	RRMS naïve	RRMS NAT	RRMS ALEM	SuS Training	SuS Prediction
CD4	PB naïve [x10^{-2}]	94±3	92±4	94±2	82±7	90±9	92±3
	CSF naïve [x10^{-5}]	10.66±26.58	55.49±96.47	4.93±14.7	71.1±136.6	18.94±26.55	n.a.
	PB diff [x10^{-2}]	6.4±3.2	6.9±3.2	5.6±0.2	164±6.9	9.5±8.6	7.6±3.4
	CSF diff [x10^{-5}]	9.1±19.9	64.3±114.5	7.04±10.2	181.1±346.8	21.2±28.3	n.a.
CD8	PB naïve [x10^{-2}]	89±7	87±8	91±5	77±10	78±18	88±8
	CSF naïve [x10^{-4}]	9.7±24.9	57.6±117.9	13.6±26.3	99.7±178.4	11.1±14.6	n.a.
	PB diff [x10^{-2}]	10.6±6.8	12.4±8.1	8.5±4.8	21.6±9.7	22.3±17.5	12.4±7.9
	CSF diff [x10^{-4}]	5.3±20.5	38.0±81.4	845.3±475.5	90.9±169.1	7.7±8.3	n.a.
B	PB naïve [x10^{-2}]	99±1	99±2	99±1	100±0	99±2	100±0
	CSF naïve [x10^{-5}]	5.1±7.9	149.7±403.3	4.8±9.3	103.7±198.5	33.0±78.0	n.a.
	PB diff [x10^{-3}]	5.1±6.2	6.9±14.1	5.4±6.8	2.8±2.1	10.7±17.7	3.9±6.9
	CSF diff [x10^{-6}]	0.01647± 0.0815	277.4±811.9	3.5±19.0	203.1±443.6	0.04048±1.4	n.a.
NK	PB naïve [x10^{-2}]	6.3±4.2	7.4±5.7	11.6±8.0	21.2±10.4	7.3±6.3	10.5±10.5
	CSF naïve [x10^{-5}]	7.9±13.5	76.2±254.1	6.8±9.2	134.6±302.9	7.8±8.7	n.a.
	PB diff [x10^{-2}]	94±4	93±6	88±8	79±10	93±6	90±11
	CSF diff [x10^{-5}]	2.2±7.6	15.0±36.8	4.5±9.2	16.6±29.9	2.9±2.8	n.a.

Supplemental Table 1: Patient based data. Mean values ± standard deviations (SD) of relative proportions of the respective lymphocyte subsets are displayed. N.a. = not applicable.
